# Induction of Cell Stress in Neurons from Transgenic Mice Expressing Yellow Fluorescent Protein: Implications for Neurodegeneration Research

**DOI:** 10.1371/journal.pone.0017639

**Published:** 2011-03-08

**Authors:** Laura H. Comley, Thomas M. Wishart, Becki Baxter, Lyndsay M. Murray, Ailish Nimmo, Derek Thomson, Simon H. Parson, Thomas H. Gillingwater

**Affiliations:** 1 Euan MacDonald Centre for Motor Neurone Disease Research, University of Edinburgh, Edinburgh, Lothian, United Kingdom; 2 Centre for Integrative Physiology, University of Edinburgh, Edinburgh, Lothian, United Kingdom; Hertie Institute for Clinical Brain Research and German Center for Neurodegenerative Diseases, Germany

## Abstract

**Background:**

Mice expressing fluorescent proteins in neurons are one of the most powerful tools in modern neuroscience research and are increasingly being used for *in vivo* studies of neurodegeneration. However, these mice are often used under the assumption that the fluorescent proteins present are biologically inert.

**Methodology/Principal Findings:**

Here, we show that *thy1*-driven expression of yellow fluorescent protein (YFP) in neurons triggers multiple cell stress responses at both the mRNA and protein levels *in vivo*. The presence of YFP in neurons also subtly altered neuronal morphology and modified the time-course of dying-back neurodegeneration in experimental axonopathy, but not in Wallerian degeneration triggered by nerve injury.

**Conclusions/Significance:**

We conclude that fluorescent protein expressed in *thy1*-YFP mice is not biologically inert, modifies molecular and cellular characteristics of neurons *in vivo*, and has diverse and unpredictable effects on neurodegeneration pathways.

## Introduction

The development of transgenic mice endogenously expressing spectral variants of fluorescent proteins such as GFP, YFP and CFP (collectively termed XFPs) in neurons, under the control of the neuronal-specific *thy1* gene, has revolutionised modern neuroscience research, facilitating experimental breakthroughs and revolutionising our understanding of the form and function of the nervous system in health and disease [1]–[8]. Early studies on *thy1*-XFP mice reported that expression of fluorescent proteins in neurons had no discernable effect on neuronal morphology or any detectable level of toxicity [1], in agreement with earlier reports suggesting that XFPs are biologically inert [9]. Thus, there is an underlying assumption that the presence of fluorescent protein has no effect on the cellular or molecular composition of neurons *in vivo*. However, neurons in these mice contain large amounts of a foreign fluorescent protein. Such fluorescent proteins are known to be capable of altering the cellular and molecular characteristics of living cells in other experimental model systems *in vivo* and *in vitro* (e.g. the cardiovascular system), triggering stress responses and pathological changes [10]–[11]. Given the ever-increasing use of *thy1*-XFP mice for not only cellular, but also molecular, experiments in the healthy and pathological nervous system, a thorough investigation of the consequences of XFP expression in neurons of these mice is required.

## Results and Discussion

**Figure 1:**
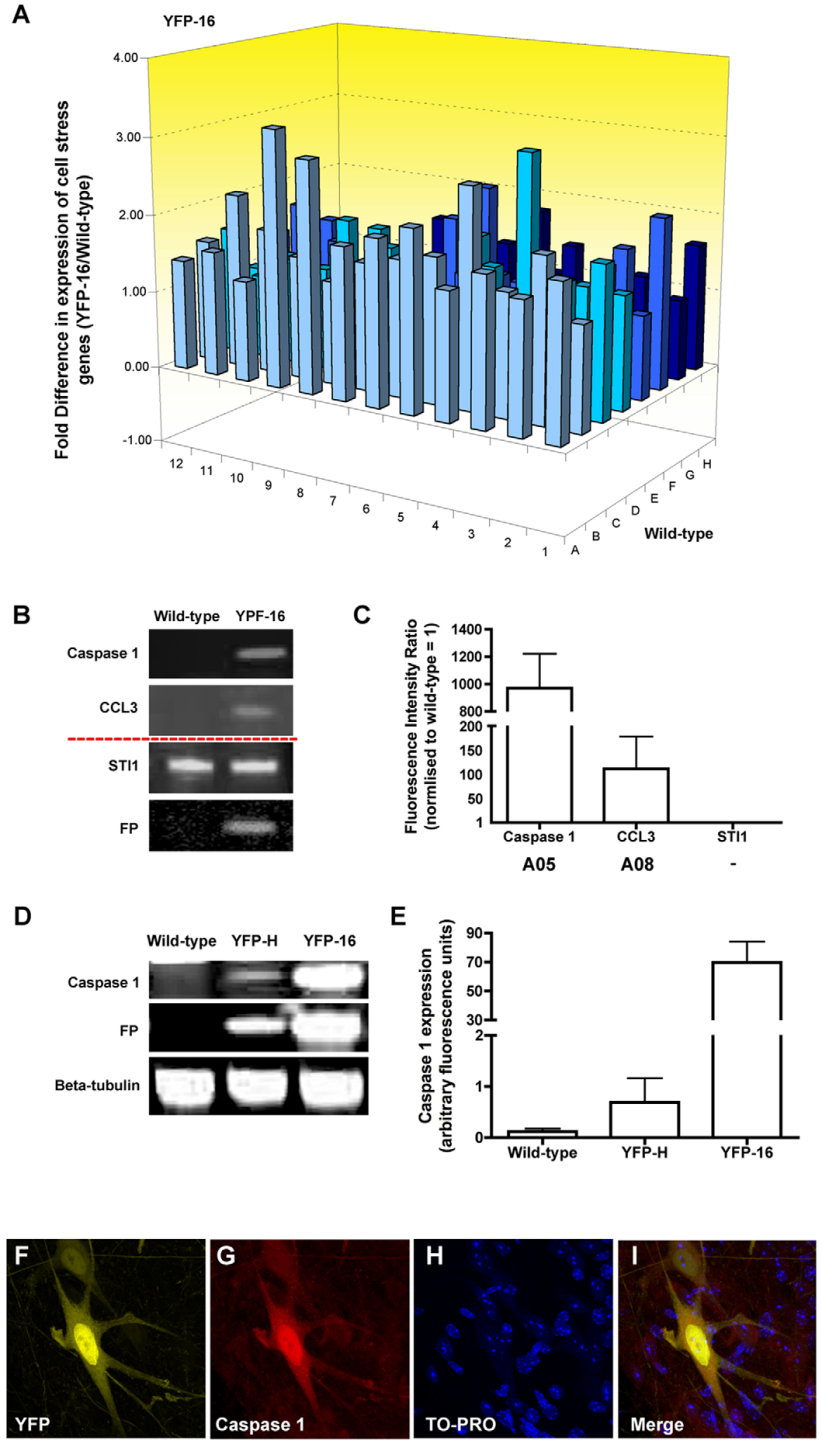
YFP expression activates cell stress responses in neurons *in vivo*. A – 3D bar chart showing fold-differences in mRNA expression levels for 84 cell stress related genes comparing spinal cord from YFP-16 mice with wild-type mice (N = 3 samples, each consisting of pooled tissue from 3 mice). Note that we only observed increased expression of cell stress related genes. B – Representative fluorescent western blots for cell stress proteins in the spinal cord of wild-type and YFP-16 mice. Both caspase 1 (Casp1) and CCL3 had increased expression in YFP-expressing tissue, whereas STI1 (a stress protein not on the array) remained at the same levels found in wild-type mice and YFP (FP) was only present in YFP-16 tissue. C - Bar chart (mean ± s.e.m.) showing quantification of protein expression levels in YFP-16 spinal cord (normalised to wild-type: fluorescence intensity ratio of 1 = identical to wild-type), confirming increased expression levels of CCL3 and caspase 1 (N = 3 samples, each consisting of pooled tissue from 3 mice). D/E - Representative fluorescent western blots and bar chart showing caspase 1 expression in the spinal cord of wild-type, YFP-H (low YFP expression) and YFP-16 (high YFP expression) mice. Increased levels of caspase 1 correlated with the amount of YFP present. F-I - Representative confocal micrographs showing caspase 1 immunohistochemistry (red = caspase 1; yellow = YFP; blue = TO-PRO) in the spinal cord of a YFP-H mouse. Increased caspase 1 immunolabelling was restricted to YFP-positive neurons. Scale bar = 20 µm.

We initially tested whether expression of yellow fluorescent protein modifies neuronal cell stress pathways in *thy1*-YFP line 16 (YFP-16) mice *in vivo*. YFP-16 mice from our breeding colony (founders originally purchased from Jackson Laboratories) appeared identical to those previously reported in the literature, expressing YFP in the vast majority of neurons in the peripheral nervous system [1]. We used cell stress pathway-specific RT^2^ profiler PCR arrays to quantify and compare expression of known cell stress-related genes in the spinal cord of *thy1*-YFP line 16 (YFP-16) mice on a C57Bl/6 background compared to wild-type C57Bl/6 controls from the same breeding colony. Surprisingly, half of all the cell stress genes (41 out of 84) on the PCR array were up-regulated more than 1.5-fold in spinal cord from YFP-16 mice (Figure 1A; Table 1). Of these, several had expression levels increased more than 2-fold, including genes involved in cell stress activation, inflammation and apoptosis (Table 1). None of the genes on the array were found to be significantly down-regulated >1.5 fold in YFP-16 mouse spinal cord (Figure 1A).

**Table 1:** Mouse SuperArray data showing greater than 1.5 fold cell stress RNA expression changes in the spinal cord of YFP-16 mice compared with wild-type controls (*array cell refers to the location on the 3D bar chart in Fig. 1A).

Array cell*	Symbol	Fold change	Role in cell stress
A09	Ccl4	3.26	Inflammation
C03	Cyp7a1	3.14	Oxidative or metabolic stress
A08	Ccl3	2.94	Inflammation
B04	Csf2	2.77	Inflammation
A05	Casp1	2.30	Apoptosis signalling
F06	Nfkbia	2.26	Apoptosis signalling
B11	Cyp2c29	2.23	Oxidative or metabolic stress
F01	Lta	2.18	Inflammation
A06	Casp8	2.11	Apoptosis signalling
B02	Chek2	2.07	DNA damage and repair
A01	Anxa5	1.95	Apoptosis signalling
A07	Ccl21b	1.94	Inflammation
C01	Cyp4a10	1.93	Oxidative or metabolic stress
G05	Tnfsf10	1.92	Apoptosis signalling
D05	Gpx2	1.90	Oxidative or metabolic stress
A03	Bax	1.89	Apoptosis signalling
B05	Cxcl10	1.84	Inflammation
D09	Hmox1	1.84	Oxidative or metabolic stress
B10	Cyp2b9	1.84	Oxidative or metabolic stress
F07	Nos2	1.79	Inflammation
D08	Gstm3	1.79	Oxidative or metabolic stress
B06	Cyp1a1	1.74	Oxidative or metabolic stress
F02	Mdm2	1.72	Growth arrest and senescence
C07	Egr1	1.72	Proliferation and carcinogenesis
C04	Ddit3	1.68	Growth arrest and senescence
F12	Rad50	1.67	DNA damage and repair
A02	Atm	1.66	DNA damage and repair
E11	Il1b	1.65	Inflammation
C02	Cyp4a14	1.64	Oxidative or metabolic stress
H01	Gusb	1.63	Oxidative or metabolic stress
B07	Cyp1b1	1.63	Oxidative or metabolic stress
G08	Ugt1a2	1.63	DNA damage and repair
C12	Fmo1	1.62	Oxidative or metabolic stress
A04	Bcl2l1	1.62	Apoptosis signalling
E02	Hspa4	1.60	Heat Shock
A11	Ccnd1	1.59	Proliferation and carcinogenesis
B03	Cryab	1.56	Oxidative or metabolic stress
B09	Cyp2b10	1.56	Oxidative or metabolic stress
B12	Cyp3a11	1.56	Oxidative or metabolic stress
F11	Rad23a	1.52	DNA damage and repair
G04	Tnfrsf1a	1.52	Apoptosis signalling

To confirm that the RNA changes observed in YFP-16 mouse spinal cord resulted in corresponding changes at the protein level, expression levels for 2 proteins selected from the PCR array (caspase 1 and CCL3) were validated in YFP-16 mouse spinal cord using quantitative fluorescent western blot (Figure 1B,C). Expression levels of another cell stress protein not included on the array - stress inducible protein 1 (STI1) – remained unchanged in YFP-16 tissue, showing that not all cell stress proteins responded to the presence of YFP (Figure 1B,C). Increased expression of caspase 1 correlated directly with the amount of YFP present, as levels in YFP-16 mouse spinal cord were 70-fold higher than levels in YFP-H mice where YFP is only expressed in a minority of neurons (Figure 1D,E) [1]. Importantly, immunohistochemistry for caspase 1 expression in YFP-H mouse spinal cord confirmed that increased cell stress responses were restricted to neuronal cells expressing YFP (Figure 1F–I). Taken together, these data demonstrate that expression of YFP in mouse neurons *in vivo* activates a robust, but selective, cell stress response at both the RNA and protein level.

**Figure 2:**
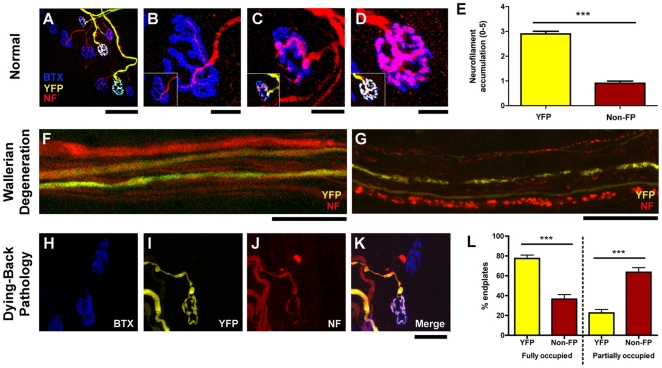
YFP expression in motor neurons subtly disrupts normal neuronal morphology and alters responses to dying-back pathology but not Wallerian degeneration. A–D – Representative confocal micrographs of NMJs in the levator auris longus muscle from a YFP-H mouse labelled for neurofilaments (NF: red) and postsynaptic motor endplates (BTX: blue). Note that all motor endplates were innervated, but only a small proportion of motor axons were YFP-positive in these mice (A). Panels B–D show high power micrographs of NMJs identified in panel A. Note abnormal accumulations of NF only in the motor nerve terminals of YFP-positive NMJs (B = NF accumulation score of 0, C = 3, D = 5). E – Bar chart (mean ± s.e.m.) showing quantification of NF accumulation in motor nerve terminals from YFP-H mice (0 = no accumulation; 5 = large abnormal accumulation), revealing a significant increase in NF accumulation in YFP-positive terminals (N = 5 mice, n = 9 muscles; *** P<0.001 Mann-Whitney test). F–G – Representative confocal micrographs of intramuscular axons supplying the transversus abdominis muscle from YFP-H mice (also labelled for NFs; red), before (F) and 20 hours after (G) intercostal nerve lesion. The presence of YFP did not alter the rate or morphological appearance of Wallerian degeneration after nerve injury, with NF fragmentation occurring in YFP-positive and –negative axons in all nerves examined (N = 6 mice, n = 6 nerves). H–K - Representative confocal micrographs of two NMJs in the levator auris longus muscle from a late-symptomatic (P24) *wasted*/YFP-H mouse labelled to reveal NFs (red) and postsynaptic motor endplates (BTX: blue). Note how the motor axon with YFP (bottom NMJ) remained intact whereas the motor axon without YFP (top) was undergoing retraction, characteristic of a dying-back pathology. L - Bar chart showing quantification of dying-back pathology at the NMJ in late-symptomatic (P24) *wasted*/YFP-H mice, revealing a significant attenuation of dying-back pathology in motor nerve terminals where YFP was present (i.e. a retention of endplates fully occupied by overlying NFs and a reduction in the numbers of partially occupied endplates; N = 4 mice, n = 7 muscles; *** P<0.001 Mann-Whitney test). Scale bars = 80 µm (A), 40 µm (B–D), 30 µm (F–G), 50 µm (H–K).

**Figure 3:**
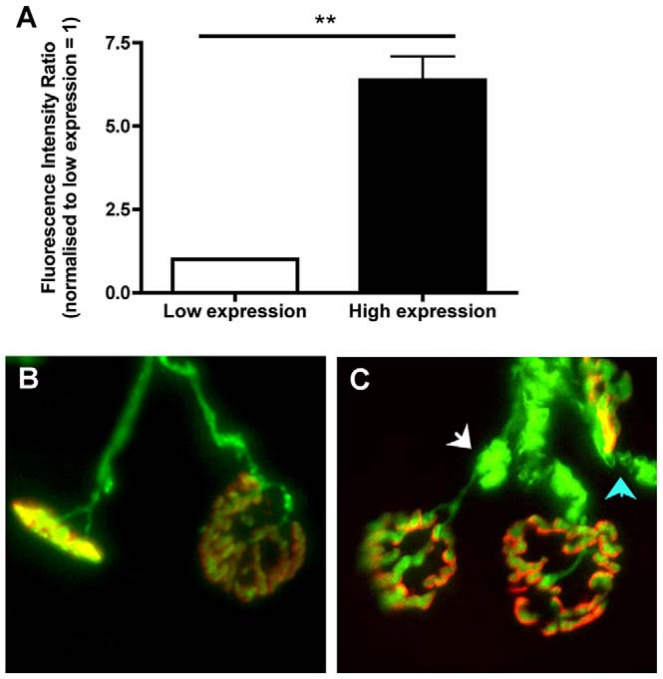
Increased incidence of morphological abnormalities at the NMJ in tissue banked from a strongly-expressing YFP-16 mouse colony. A - Bar chart (mean ± s.e.m.) showing relative expression levels of YFP in the sciatic nerve of YFP-16 mice from the current Edinburgh colony (low expression), compared to YFP levels in tissue banked from a now defunct high-expressing YFP-16 colony previously held at the University of Leeds, quantified using fluorescent western blot (** P<0.01 unpaired two-tailed t test). B–C - Representative micrographs showing NMJ morphology in low-expressing YFP-16 mice (B) compared to high-expressing YFP mice (C). Motor endplates are labelled and shown in red. Note the presence of large YFP accumulations in both axons (white arrow) and motor nerve terminals (blue) from the high-expressing tissue. Such morphological abnormalities were present in all preparations examined from high-expressing YFP mice.

We next sought to establish whether activation of cell stress pathways in YFP-expressing neurons had any adverse effects on neuronal morphology. We examined lower motor neurons innervating the levator auris longus muscle in YFP-H mice, allowing us to directly compare the morphology of YFP-expressing neurons with neighbouring non-expressing neurons in the same muscles from individual animals (Figure 2A). As previously reported [1], all neuromuscular junctions in 2–4 month old YFP-H mice were fully innervated by a single motor axon collateral (Figure 2A). However, higher-resolution analysis revealed a significant increase in abnormal accumulations of neurofilaments in distal axons and motor nerve terminals when YFP was present. This morphology is similar to cytoskeletal changes considered to represent early pathological changes in neurons [3], [12] (Figure 2B–E). Neurofilament accumulations greater than a score of 1 on our 0–5 accumulation ranking scale were very rarely observed in distal axons and motor nerve terminals innervating the entire levator auris longus muscle in wild-type mice (data not shown). This reduces the likelihood that the neurofilament accumulations we observed in YFP-expressing neurons reflected an underlying biological diversity in neurofilament distribution between motor neuron pools that may or may not be selectively labelled in YFP-H mice. Retrospective examination of banked tissue from a now defunct YFP-16 mouse colony with very high YFP expression levels (∼7 times greater than those found in our standard YFP-16 colony) revealed more severe morphological abnormalities, suggesting a dose-dependent effect (Figure 3).

It has previously been suggested that modulation of cell stress pathways can alter the response of neurons to neurodegenerative stimuli [13]–[14]. Therefore, we next addressed whether the presence of YFP in neurons also affected neurodegeneration pathways *in vivo*. YFP protein persists and retains its fluorescent properties in degenerating neurons, and has previously been used to visualise the breakdown of axons and synapses in a range of different models of neurodegeneration [3]–[5], [15]. The rapid instigation (within 20 hours of nerve lesion), and morphological correlates, of Wallerian degeneration following nerve lesion were not altered in YFP-expressing motor neurons (Figure 2F–G). However, in *wasted* mice, where loss of the translation elongation factor *eEF1A2* causes a severe distal axonopathy [15], the majority of YFP-expressing neurons remained intact whilst neighbouring non YFP-expressing neurons were in the final stages of degeneration, even at late-symptomatic ages (Figure 2H–L). Therefore, the severity of dying-back pathology, which is mechanistically distinct from Wallerian degeneration [15], was significantly modified in YFP-expressing motor neurons from *wasted* mice.

In conclusion, our finding that expression of YFP in neurons instigates cell stress pathways means that fluorescent protein expressed in neurons from *thy1*-YFP mice is not biologically inert. This finding provides importance evidence, supported by data from other *in vivo* and *in vitro* experimental systems [10]–[11], [16]–[17], that fluorescent proteins expressed in cells/tissues can modify their molecular composition and cellular activities. Care must now be taken with regard to experimental design and data interpretation, particularly when investigating molecular mechanisms of neurodegeneration in mice endogenously expressing fluorescent protein in neurons. Future studies must take into account the modified molecular characteristics and altered phenotypic responses observed here and perform necessary controls to ensure that any responses they are reporting are a direct result of experimental intervention rather than simply a consequence of the expression of fluorescent protein.

## Materials and Methods

### Ethics statement

All animal experiments were approved by a University of Edinburgh internal ethics committee and were performed under license by the UK Home Office (project license number 60/3891).

### Mice

Breeding colonies of *thy1*-YFP-H mice [1] and *thy1*-YFP-16 mice, both on a C57BL/6J background, were originally purchased as breeding pairs from Jackson Laboratories and established and maintained in animal care facilities (standard SPF conditions) at the University of Edinburgh. Wild-type C57Bl/6J mice (not expressing YFP protein) from the same breeding colony were used for controls throughout. Mice were sacrificed by inhalation of isofluorane (2% in 1∶1 N_2_O/O_2_). All procedures were carried out under licensed authority from the UK Home Office. The YFP status of mice was ascertained by examining ear punches for YFP-positive neurons. To determine the effects of YFP expression on dying-back pathology, YFP-H mice were crossed with *wasted* mice [15]. *Wasted*/YFP-H mice were genotyped using standard PCR techniques, as described previously [15], and were taken for analysis at post-natal day 24 (late-symptomatic). The banked tissue from a previous, now defunct, colony of strongly-expressing *thy1*-YFP-16 mice was obtained from mice originally supplied to the University of Leeds by Jackson Laboratories.

### Cell stress array

Mouse cell stress focused pathway arrays in a 96-well plate format (PAM-003A), compatible with an ABI 7000 real-time PCR machine, were used to assay cell stress gene expression (3 comparisons for YFP-16 and 3 for wild-type controls, each comparison composed of pooled whole spinal cord tissue from 3 individual 2 month old mice). RNA was extracted as previously described [14]. Samples were added to the reaction plate and signal amplification by PCR was carried out using Sybr-Green ‘1 step qRT-PCR kit’ (Invitrogen). Analysis was carried out using the Analysis Suite spreadsheet provided by Tebubio SuperArrays. Gene functions listed in Table 1 were obtained from the SuperArray product specification sheets.

### Quantitative fluorescent (Li-COR) western blots

Total protein was isolated from spinal cords and quantitative western blots were performed as described previously [18]–[20]. Primary antibodies were used as per manufacturers instructions (STI1 – BD Biosciences; Caspase 1 and CCL3 – Abcam; GFP - Millipore). Odyssey secondary antibodies were used in accordance with manufacturers instructions (Goat anti mouse IRDye 680 and Goat anti rabbit IRDye 680). Blots were imaged using an Odyssey Infrared Imaging System (Li-COR Biosciences). Quantification was performed on single channels with the analysis software provided. Bands were identified according to their molecular weight and the arbitrary fluorescence intensity was calculated by Odyssey software.

### Spinal cord immunohistochemistry

Entire vertebral columns were removed and immediately immersed in 4% paraformaldehyde solution for 1 hour. Spinal cords were removed from the vertebral column and fixed for a further 30 mins before embedding in 2% low-melting point agarose. Embedded spinal cords were mounted on a vibratome and transverse sections cut at a thickness of ∼200 µm for immunohistochemical staining. Sections were blocked in 4% bovine serum albumin (BSA) and 1.5% TritonX in 0.1 M PBS for 30 minutes before incubation in primary antibodies directed against caspase 1 (1∶500 dilution; Abcam) overnight. After washing for 2 hours in 0.1 M PBS, sections were incubated for 2 hours in a 1∶30 dilution of swine anti-rabbit TRITC-conjugated secondary antibody (Dako). Sections were co-stained with the nuclear dye TOPRO-3 (Molecular Probes), whole-mounted in Mowoil® (Calbiochem) on glass slides and cover-slipped for subsequent imaging.

### Surgery

Mice were anaesthetised by inhalation of halothane (2% in 1∶1 N_2_O/O_2_) before exposing the intercostal nerves innervating the transversus abdominis muscle. Nerves were cut before suturing the skin and allowing the mouse to recover for 20 hours. Post-operative mice were maintained in standard animal house conditions.

### Immunohistochemical analysis of axons and neuromuscular junctions

The levator auris longus (LAL) [21]–[22] and/or transversus abdominis (TVA) [12] muscles and their nerve supply were dissected and fixed in 0.1 M PBS containing 4% paraformaldehyde (Electron Microscopy Science) for 10 minutes. YFP tissue was additionally double-labelled by exposure to α-bungarotoxin conjugated to Alexa Fluor® 647 (Alexa 647-α-BTX; 5 mg/ml, Invitrogen) for 30 minutes to label post-synaptic acetylcholine receptors. Muscles were blocked in 4% bovine serum albumin (BSA) and 1.5% TritonX in 0.1 M PBS for 30 minutes before incubation in primary antibodies directed against 145 kDa neurofilament proteins (1∶300 dilution; Millipore) overnight to label axons and nerve terminals. After washing for 2 hours in 0.1 M PBS, muscles were incubated for 4 hours in a 1∶30 dilution of swine anti-rabbit TRITC-conjugated secondary antibody (Dako). Muscles were whole-mounted in Mowoil® (Calbiochem).

### Microscopy

Fluorescently labelled axon and nerve/muscle preparations were viewed using a laser scanning confocal microscope (63× objective; 1.4NA; Zeiss LSM 710). Confocal Z-series were merged using Zeiss software and images assembled for analysis using Adobe Photoshop. All images were taken using sequential laser capture and with the excitation/emission spectra set to ensure no bleed-through between channels. A minimum of 50 neuromuscular junctions, selected at random, were assessed in each muscle preparation. Quantitative analysis was performed on micrographs with the YFP channel initially excluded, so that the operator did not know the YFP status of the tissue being analysed. For analysis of neurofilament accumulation in normal tissue, endplates were qualitatively assigned a score between 0 and 5, with 0 representing healthy neuromuscular junctions with no evidence of neurofilament accumulation and 5 representing nerve terminals with large abnormal accumulations of neurofilament. The YFP channel was revealed subsequent to quantification to determine the YFP status of each nerve terminal. For occupancy counts in *Wasted/*YFP-H mice undergoing dying-back pathology, endplates were categorised as either partially occupied (neurofilament partially overlying the endplate) or fully occupied (neurofilament entirely overlying the endplate). Vacant endplates were excluded from analysis since there was no way to establish their original YFP status.

### Statistical analysis

All data were collected into Microsoft Excel spreadsheets and analysed using GraphPad Prism software. All bar charts shown are mean ± s.e.m. Statistical significance was considered to be p<0.05 for all analyses. Individual statistical tests used are detailed in figure legends.
